# Methods used to estimate the size of the owned cat and dog population: a systematic review

**DOI:** 10.1186/1746-6148-9-121

**Published:** 2013-06-19

**Authors:** Martin J Downes, Rachel S Dean, Jenny H Stavisky, Vicki J Adams, Douglas JC Grindlay, Marnie L Brennan

**Affiliations:** 1 Centre for Evidence-based Veterinary Medicine, School of Veterinary Medicine and Science, The University of Nottingham, Sutton Bonington Campus, LE12 5RD, Loughborough, UK; 2 PO Box 80 Mildenhall, Suffolk, IP28 9BF, UK

**Keywords:** Cat, Dog, Population estimation, Demographics, Systematic review, Epidemiological methods

## Abstract

**Background:**

There are a number of different methods that can be used when estimating the size of the owned cat and dog population in a region, leading to varying population estimates. The aim of this study was to conduct a systematic review to evaluate the methods that have been used for estimating the sizes of owned cat and dog populations and to assess the biases associated with those methods.

A comprehensive, systematic search of seven electronic bibliographic databases and the Google search engine was carried out using a range of different search terms for cats, dogs and population. The inclusion criteria were that the studies had involved owned or pet domestic dogs and/or cats, provided an estimate of the size of the owned dog or cat population, collected raw data on dog and cat ownership, and analysed primary data. Data relating to study methodology were extracted and assessed for biases.

**Results:**

Seven papers were included in the final analysis. Collection methods used to select participants in the included studies were: mailed surveys using a commercial list of contacts, door to door surveys, random digit dialled telephone surveys, and randomised telephone surveys using a commercial list of numbers. Analytical and statistical methods used to estimate the pet population size were: mean number of dogs/cats per household multiplied by the number of households in an area, human density multiplied by number of dogs per human, and calculations using predictors of pet ownership.

**Conclusion:**

The main biases of the studies included selection bias, non-response bias, measurement bias and biases associated with length of sampling time. Careful design and planning of studies is a necessity before executing a study to estimate pet populations.

## Background

There has been considerable research carried out in a variety of settings examining pet demography and the roles pets play in human life [1-6]. Pet ownership has been associated with various advantages for humans, such as decreased risk of cardiovascular disease [7], reduced doctor visits [8,9], reduced loneliness [10] and provision of emotional support [11]. Disadvantages such as trauma from accidents [12] or bites/attacks [13], allergies [14,15] and zoonotic disease [16,17] have also been associated with pet ownership. Numerous studies have been published in an effort to understand pet demographics and how to address the above issues relating to owned dogs and cats [18-21]. Corresponding government legislation has been created in many countries to help control owned dogs and provide adequate welfare for owned pets. Examples of this type of legislation can be seen in the UK [22,23], Ireland [24] and the USA [25].

In order to help understand the magnitude of these issues, a baseline animal population estimate is necessary. This baseline is especially needed when seeking to ascertain and interpret prevalence data for dog and cat diseases, to determine where animals are situated geographically and to identify the numbers of animals at risk for exotic disease outbreaks such as rabies [19,21]. These population data are also useful for the veterinary industry, as they enable more focused strategies for providing veterinary care [1]. The pet food, pharmaceutical and pet accessories industries are also interested in knowing where to focus their marketing strategies, and demographic information can aid this [26].

Many studies collecting pet population data use cross sectional study designs and administer questionnaires to a sample from a given population within the specified study regions by telephone [5,27-29], postal addresses [30], door-to-door sampling [4,5,19] or census-type selection methods [6,31]. When calculating the estimated population size of dogs and cats, studies have used methods ranging from relatively simple calculations, multiplying human numbers by dog ownership [1,5], to more complex probability estimates [3] or statistical models [32].

There have been discrepancies in the estimated number of dogs and cats found in the same geographical area at the same period of time, depending on the sampling and the calculation methods used. In a UK study [3], the estimated sizes of the owned cat and dog populations in 2006 were 10,332,955 cats (95% CI: 9,395,642 to 11,270,269) and 10,522,186 dogs (95% CI: 9,623,618 to 11,420,755), whereas a survey carried out by the Pet Food Manufacturers Association (PFMA) estimated the cat and dog population at just over 7 million each for the same year [26]. Given that there are a number of different methods that can be used leading to varying population estimates, the aim of this systematic review was to evaluate the methods that have been used for estimating the size of owned cat and dog populations and to assess the biases associated with these methods.

## Results

### Bibliographic databases

When the searches from all the bibliographic databases were combined, 135824 records were obtained. A total of 71713 duplicate records were found in the combined dataset, leaving 64111 papers to be considered for inclusion in the analysis. After title and abstract screening, 152 relevant papers were obtained for full paper screening. There were 29 non-English language papers that needed to be translated. Of the 38 papers requested through British Library Document Supply Centre, 12 papers could not be obtained. Five papers were found to be based on the data from previously published studies, and were removed.

**Figure 1:**
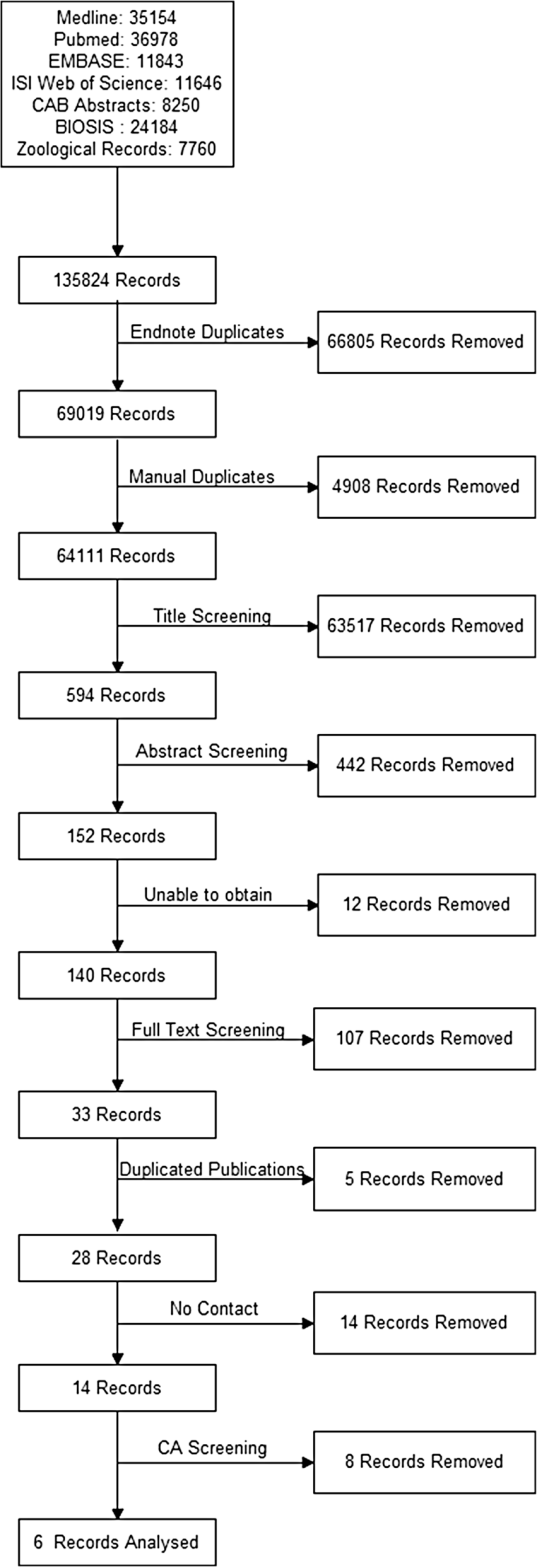
Flow diagram showing the total number of records identified and the number of records filtered at each stage of the selection process from the literature search of a systematic review on pet population estimation.

During full text screening, two papers required assessment from the third reviewer and a total of 28 studies met all the initial inclusion criteria and didn’t meet the exclusion criteria as shown in the flow chart in Figure 1. Of these 28 studies, 14 papers had insufficient details about the methods of pet number estimation to enable them to be repeated and required additional information from the authors. Only two authors responded with sufficient methodological information to enable the studies concerned to go forward for critical appraisal and only six studies remained in the final analysis after critical appraisal (Table 1).

**Table 1:** Outcome of author contact and critical appraisal (CA) of 31 studies that satisfied the initial inclusion criteria in a systematic review examining methods to estimate owned dog and cat populations

**Literature identified**^**1**^	**Clear methods**^**2**^	**Result of attempt to contact author**^**3**^	**Include after critical appraisal**
**Literature Searches**			
Acosta-Jamett et al. [33]	Yes		No
Agostini et al. [34]	No	No author found	
Agostini et al. [35]	Yes		No
**AVMA**[36]	**Yes**		**Yes**
Brooks [37]	No	No author found	
**Butler and Bingham**[19]	**Yes**		**Yes**
De Balogh et al. [38]	Yes		No
Degregorio et al. [39]	No	No author found	
Dias et al. [40]	No	Author responded	No
Egenvall et al. [41]	No	No author found	
Gregory and Reid [42]	No	No author found	
Griffiths and Brenner [43]	Yes		No
Ibarra et al. [44]	Yes		No
Kitala et al. [45]	Yes		No
Larrieu et al. [46]	No	No author found	
**Lengerich et al.**[47]	**Yes**		**Yes**
Martin et al. [48]	No	No author found	
Morales et al. [49]	No	No author found	
**Murray et al.**[3]	**No**	Author responded	**Yes**
Matter [21]	No	No author found	
Okoh [50]	No	No author found	
**Ortega-Pacheco et al.**[51]	**Yes**		**Yes**
Patronek et al. [52]	No	No author found	
Rangel et al. [53]	No	No author found	
Rautenbach et al. [54]	No	No author found	
Serafini et al. [4]	No	No author found	
**Slater et al.**[5]	**Yes**		**Yes**
Subbaraj et al. [55]	Yes		No
**Google Searches**			
**AVMA**[1]	**Yes**		**Yes**
Pet Plan [56]	No	No author found	
PFMA [26]	No	Author responded	No

1. Studies in bold were included in the final review.

2. Methods were judged to be clear (‘yes’) when the methods for calculating the population size estimate reported in the study were transparent and repeatable.

3. Where the methods were clear enough, no attempt was made to contact the author.

During the critical appraisal process one study required assessment from the third reviewer (RD). In the eight studies that were excluded during the critical appraisal process there were particular problems common to most of them: all studies had insufficient description of their methods for them to be repeated, and for five of the studies it was difficult to ascertain if the selection process would lead to a sample of participants that was representative of the target population. Only one study used sample size justification in the development of the study. None of the eight excluded studies supplied information about non-responders and only two studies had undertaken measures to address non-responders in the methods. None of the studies discussed selection biases or any limitations of the study.

Issues relating to the studies that were included in the review after critical appraisal were: five studies had insufficient description of their methods for them to be fully repeated and for four of the studies it was difficult to ascertain if the selection process would lead to a representative sample of the target population. Only three studies used sample size estimations in the development of the study. Three of the studies supplied information about non-responders and only one study had undertaken measures to address non-responders in the methods. Five of the studies discussed selection bias in their study.

### Google

**Figure 2:**
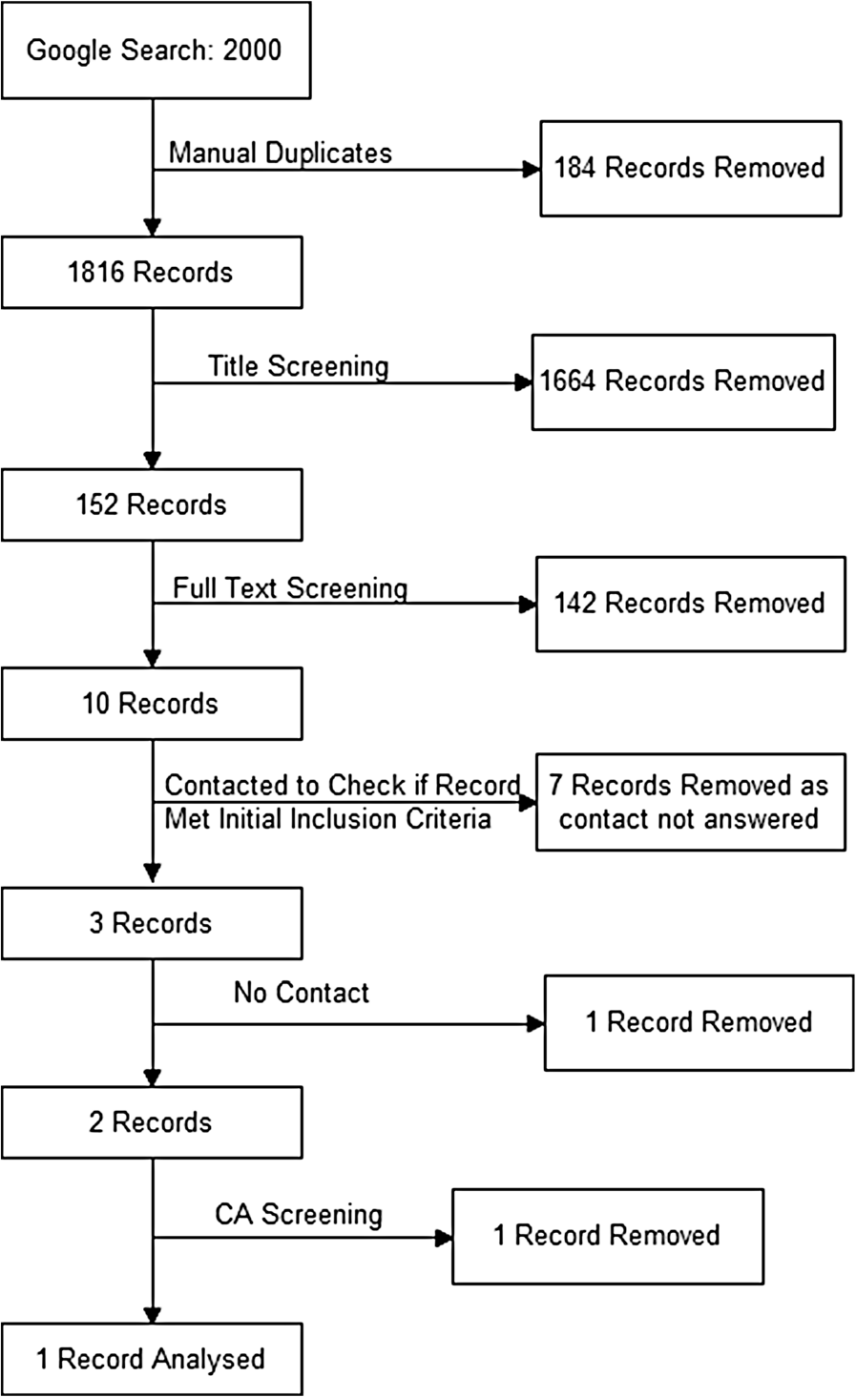
Flow diagram showing the total number of records identified and the number of records filtered at each stage of the selection process of a Google search carried out as part of a systematic review on pet population estimation.

When the search from Google was completed 2000 records were extracted from the search engine. A total of 184 duplicate records were found leaving a total of 1816 records to be examined (Figure 2). Of these 1816, only 152 remained for full analysis after title screening for relevance. After applying the inclusion and exclusion criteria (one website required assessment by the third reviewer [57]), only ten web pages remained. An attempt was made to contact the website authors in seven cases where it was difficult to identify if the content of the web page met the inclusion criteria and in two others where the methods were not clear enough to repeat the estimation of the population. None of the website authors responded sufficiently for them to be included in further analyses (Table 1). Only one web page remained after the critical appraisal stage, and was included in the final analysis after appraisal (Figure 2).

Two of the included studies were carried out by the same association ten years apart and the second study was modified based on previous studies [1,36]. One of these studies was identified in the bibliographic databases [36] while the other was identified through the Google search [1].

### Sampling/selection methods

Two tables summarising the findings relating to the collection and analytical/statistical methods were created for each included study (Tables 2 and 3). The methods used for collecting data were mail-out surveys using a commercial list of contacts [1,36], door-to-door surveys [19,51], random digit dial telephone surveys [47,51] and randomised telephone surveys using a commercial list of numbers [3,5]. None of the included studies used specific definitions for pet/owned cat or dog, but asked the respondent if they owned a dog or cat.

**Table 2:** Characteristics of the seven studies included in the final analysis of a systematic review examining methods to estimate owned dog and cat populations

**Paper**	**Aim**	**Data collection method**	**Setting**	**Calculations**
AVMA [36]	No aim stated.	Questionnaire mailed to a random selection of potential participants from a list supplied by a commercial company.	National population of the United States of America	Mean number of pets per pet owning household multiplied by (the proportion of pet owning households from the survey by the number of households from National statistics).
				Dogs = 52.9 million
				Cats = 59.1 million
AVMA [1]	No aim stated.	Questionnaire mailed to a random selection of potential participants from a list supplied by a commercial company.	National population of the United States of America	Mean number of pets per pet owning household multiplied by (the proportion of pet owning households from the survey by the number of households from National statistics).
				Dogs = 69.926 million
				Cats = 74.059 million
Butler and Bingham [19]	To provide baseline data on the demography and ecology of the dog population in communal lands in Zimbabwe.	Door to door survey.	National population of Zimbabwe. Seven communal lands surveyed: Ngorima, Soswe, Kandeya, Gokwe, Tsholotsho, Dande, Mtetengwe	Average number of dogs per capita from the survey multiplied by the number of people in Zimbabwe from human statistics.
				Dogs = 1.36 million
Lengerich et al. [47]	To apply a random-digit dial telephone survey method for estimating the owned canine and feline populations, and estimate the proportion of dogs and cats with cancer.	Random-digit dial telephone survey.	Population of Marion and Tippecanoe counties of Indiana.	Total number of dogs and cats from the survey by the inverse of the sample fraction from the human census.
				Cats:
				Marion = 94,998 (74,348 to 115,648)
				Tippecanoe = 17,165 (12,569 to 21,761)
				Dogs:
				Marion = 144,039 (121,55 to
				166,523)
				Tippecanoe = 18,000 (14,445 to 21,555)
Murray et al. [3]	To identify characteristics of dog-owning and cat-owning households from a large cross-sectional study and to use these data to estimate the size of the dog and cat populations in the UK, using a method that could easily be repeated to enable pet ownership trends to be monitored.	Telephone survey of a random selection of telephone numbers from a commercially available list of numbers.	National population of the United Kingdom	The predicted cat and dog numbers for each category in the size of the household by the location were calculated from logistic regression of the data from the survey and multiplied by the number of households within each category from national statistics.
				Cats = 10,332,955 (9,395,642 to 11,270,269)
				Dogs = 10,522,186 (9,623,618 to 11,420,755)
Ortega-Pacheco et al. [51]	To generate information regarding the size and structure of the owned-dog populations, and learn about people’s opinions about their dogs and how they take care of them in three rural areas and a large city of Yucatan, Mexico.	Random-digit dial telephone survey in one large urban area and door to door survey in three rural areas.	Three rural areas (Molas, Dzununczn and San Jose Tzal) and one urban area (Merida city) in Yucatan state, Mexico.	Mean number of dogs per household from the survey multiplied by the number of households from National statistics.
				Molas = 568.5
				Dzununczn = 560
				San Jose Tzal = 844.5
				Merida = 1163
Slater et al. [5]	To document the owned pet population size and type including reproduction and dog registration.	Telephone survey of a random selection of telephone numbers from a commercially available list of numbers.	Province of Teramo, Italy	Mean number of pets per pet owning household by the proportion of pet owning households from the survey multiplied by the number of households from National statistics.
				Cats = 37,081
				Dogs = 67,183

**Table 3:** Advantages and disadvantages of four different collection methods that were used in the seven studies included in the final analysis of a systematic review examining methods to estimate owned dog and cat populations

**Data collection method used to determine pet population estimate**	**Studies**	**Advantages**	**Disadvantages**
Mail out survey using a commercial list of contacts	AVMA [1,36]	Reduces bias towards wealthier participants associated with telephone surveys.	Selection bias introduced as households that are not on the commercial list are excluded. May introduce measurement bias as the participant will be aware what the study is about.
			Overestimation of population may be introduced as a period prevalence is measured in these studies.
Door to door survey	Butler and Bingham [19]	Reduces non-response.	Costly and time consuming, probably only feasible in a small study area.
			Selection bias may have been introduced as only houses that were within 500 meters of a road were included and only roads that were passable by vehicle were used. Also true random selection was not used.
	Ortega-Pacheco et al. [51]		Costly and time consuming, probably only feasible in a small study area.
			Selection bias may have been introduced in this study as only households with a telephone could be included, this may have led to households with a higher SEC being over represented. Random selection was not used in the door to door surveys.
Random-digit dialled telephone survey	Lengerich et al. [47] Ortega-Pacheco et al. [51]	Cost effective and logistically allows a large number of participants to be recruited in a short period of time.	Large numbers of non-domestic based numbers may be included leading to greater non-response.
			Selection bias may have been introduced in this study as only households with a telephone could be included; this may have led to households with a higher social economic class (SEC) being over-represented.
Randomised telephone survey using a list of numbers	Slater et al. [5]	Cost effective and logistically allows a large number of participants to be recruited in a short period of time. Reduces number of non-household based numbers associated with random digit dial surveys.	Selection bias may have been introduced in this study as only households with a telephone could be included, and if the telephone number was not listed it could not be included.
			An explanation of the study was given at the start of the interview, which may lead to measurement bias as households with pets might be more likely to complete the questionnaire.
	Murray et al. [3]		Selection bias may have been introduced in this study as only households with a telephone could be included, and if the telephone number was not listed it could not be included.

### Risk of bias

The entire set of cross sectional studies examined in the review possessed some risk of selection bias. Measurement biases may have been introduced in all the studies resulting from misclassification of pet owners, i.e. being classed as non-owners when they were pet owners due to answering the questionnaire incorrectly [1,3,5,19,36,47,51]. Some studies were prone to non-response bias where an over-estimation of the pet population could have been introduced [1,5,36]. Bias may be present due to the length of time it took to collect the data as some studies took up to six months to collect data [3] and others collected data from a previous year [1,36] giving a period prevalence of ownership rather than a point in time estimation. Other studies failed to mention a time-frame for data collection [5,47,51]. The different collection/selection methods had different advantages over each other. For example, mail-out surveys [1,36] had the potential to include all households, whereas the telephone surveys [3,5,47] only included households with telephones. The advantages and disadvantages of the different methods, including biases, are presented in Table 3.

### Methods used to estimate pet population

The statistical/mathematical methods used to determine pet populations in the included studies were mean number of dogs/cats per household multiplied by the number of households in the area [1,19,36,47,51], human density in the area multiplied by number of dogs per human [5] and calculations using regression coefficients for predictors of ownership to calculate mean pet per person figures [3]. Only Murray et al. [3] used precision measures, providing 95% confidence intervals for their estimates. The advantages and disadvantages of the statistical/mathematical methods in the included studies are presented in Table 4.

**Table 4:** **Advantages and disadvantages of the three different analytical**/**statistical methods that were used to estimate the pet population in the seven studies included in the final analysis of a systematic review examining methods to estimate owned dog and cat populations**

**Analytical/statistical methods used to determine pet population**	**Which studies used this method**	**Advantages**	**Disadvantages**
Mean number of dogs/cats per household multiplied by the number of households in the area	Lengerich et al. [47] AVMA [1,36] Butler and Bingham [19] Ortega-Pacheco et al. [51]	Simple method that does not require complex statistics. Does not rely on large sample sizes.	Prone to selection and measurement biases.
Human density multiplied by number of dogs per human	Slater et al. [5]	Simple method that does not require complex statistics. Does not rely on large sample sizes.	Prone to selection and measurement biases.
Calculations using predictors of ownership	Murray et al. [3]	Improves precision of the population estimates.	Requires large numbers of participants so may be more costly. Can be prone to measurement bias.

## Discussion

Only seven studies involving five countries were included in this review, each conducted using different methodologies with a certain level of bias. It is difficult to select an ideal method for estimating pet populations given the differences and risk bias for each method. When interpreting results of population estimates, it is important to take into account the biases and limitation of the studies and to adjust decisions based on those results.

### Biases in studies

#### Selection bias

As the cross sectional studies were subject to selection bias, it is difficult to determine if the estimates are generalisable to the population being studied [58,59]. As the different studies in this review used different methods, different levels and types of biases could be introduced in each study.

The use of telephone surveys can result in a bias towards households of higher socio-economic standing [60]. There have also been issues with declining response rates in the past, and an increase in refusals once contacted [61]. These issues may be due to a number of factors, such as increased contact via telephone for sales and marketing, and households having access to call screening/caller identification [61-64]. The majority of the studies based on telephone surveys included in this review did little to manage the potential biases [5,47,51]. However, one study tried to address this issue by using probability measures of a variable statistically associated with the number of pets in a household [3]. This can be difficult to accomplish, as finding a variable that is associated with the number of pets owned by a household/person that can be matched to human population studies can be problematic and may require a large number of participants. Also of note is a decreasing number of households using landline numbers due to the use of mobile phones as their sole telephone contact that may increase the biases associated with telephone surveys. In the USA the percentage of adults with a landline telephone had declined to 63.8%, in 2011 and 93.9% of adults without a landline had mobile phone service in the household [65].

Selection bias can be introduced in postal studies, especially where a commercial list is used for the sampling frame. Often it is difficult to be certain how the list has been compiled, and how the people on these lists compare to the target population. The studies that used mail out surveys in this review [1,36] did try to address this problem by using quota control cut-offs in order to select an overall representative population for analyses which could help to remove selection bias. However, this method makes it difficult to measure confidence intervals accurately and make inferences [66].

Selection bias can be introduced in door-to-door surveys as logistical problems can make it difficult to access participants. Butler and Bingham [19] were unable to physically access households in their study area as roads to these households were impassable or did not exist. Households away from passable roads may be more likely to own a dog for protection, or may be less likely to own a dog because they are less well-off and have less disposable income; there is no way of knowing without sampling these households. If there is little known about this un-sampled population it is difficult to adjust the analysis to take these biases into account.

When random selection is not used or the selection process is not truly random, a non-representative sample may be selected from the sample frame, as not every household has an equal chance of being selected into the study. The use of proper random selection seemed to be a particular problem in the door-to-door sampling studies, and rather than using geographical random selection or a systematic random sampling approach (starting with a random household and sampling systematically from there) they selected every second household for sampling [19] or a specific number from each street [51] giving no indication as to how the first household was selected.

#### Non-response bias

Non-response bias may occur for a number of reasons, including failure to locate or contact a household, refusal to participate/complete a questionnaire, refusal to answer specific questions or inability to communicate [67].

Mail-out surveys by their nature give the participant the time to scan through the questionnaire. A potential participant can therefore be aware that the survey is about pet ownership, and if they do not have a pet, they may be inclined not to complete the survey, thinking the survey does not apply to them. The non-responding, non-owner would not be represented in the sample, leading to over-estimation of the pet population and this may have occured in surveys of the American Veterinary Medical Association [1,36]. Non-response in mail-out surveys can be reduced using numerous methods including monetary incentives, short questionnaires, sending personalised questionnaires and letters, recorded/first class delivery, stamped return envelopes, contacting participants before sending the questionnaire, follow up contact once a questionnaire is sent, and providing non-respondents with a second questionnaire [68]. None of these methods were stated as being used by the two postal surveys in this review [1,36].

At the start of the telephone conversation, Slater et al. [5] informed the participants about the topic before the questionnaire was administered, and it is possible that participants might have been inclined to hang up if they did not have pets, thinking the survey did not apply to them. Non-response in telephone surveys can be minimized by dispatching personally addressed introductory letters in advance, increasing the number of call attempts, targeting call times and declaring credentials of the institute at the beginning of the telephone call [69-71]. Using introductory letters in advance should be used with care as while this approach may increase response rates it may also increase non-response bias as explained previously. Some telephone studies [3,47] used repeated call attempts to decrease non-response and Murray et al. [3] introduced their study stating that they were interested in those that did not own a pet as well as those who did. However other studies did not state any methods to reduce non-response bias [5,51].

#### Measurement biases

Some of the reviewed studies may have ended up with an underestimation of the population size. Pet owners may be inclined to say they did not have a pet for fear of retribution where households are not permitted to own a pet by a landlord [1,3,5,36,47,51], or a household may have a breed that is banned [3]. Methods of preventing measurement bias would be to adequately trial the questionnaire; this ensures that it is accurately measuring the outcomes of interest [72]. It is difficult to ascertain if any of the reviewed studies addressed measurement bias, as while some stated they used introductions [3,5], none stated whether anonymity was guaranteed.

#### Biases introduced by length of sampling time

In owned dog and cat population estimation, if a study takes a significant period of time to collect data it can interfere with the accuracy of the results of the estimate. This is highly dependent on the stability and growth rate of the population under investigation. For example if the birth rate is much higher than the death rate in a population, it could lead to an overestimation of the population. It is also important to note that some studies used a period prevalence of one year [1,36], rather than point estimation. A study using a period prevalence could lead to a higher population estimate than those estimating point prevalence, making it difficult to compare results.

### Quality of the studies

Overall the quality of the studies was deemed questionable, as only seven studies out of 15 were of the desired level for inclusion during the critical appraisal stage. This shows the importance of using critical appraisal tools in evidence-based veterinary medicine as a means of assessing quality both for inclusion in a review and for use in practice. It is important to note that the studies generally failed in areas that are critical for cross sectional studies such as; having a clear research aim, complete transparent description of the methods used, sample size justification/power analysis [73], and collecting and addressing information about non-responders and selection bias [59,74] This also highlights the importance of reporting guidelines in increasing the transparency of research and these should be used in the development and reporting of scientific papers [75]. This study demonstrates that while there is a reporting guideline for observational studies (the STROBE statement [76], and previous to this, recommendations for survey reporting [73]), authors and reviewers of published studies seem either unaware of them or are unwilling to use them, as has also been demonstrated by Cobo et al. for a range of study types [77].

The ideal method for determining the pet population of an area would be to do a complete census of the area, including all households and pets. If this is not feasible, the households should be selected at random from a sample frame that includes all households in the given area. If the sample frame cannot be taken from a suitable population, or selection is likely to lead to biases, then appropriate statistical methods should be used to help deliver a more precise estimate of the population. If there is a linear association between the number of owned cats or dogs and the independent variable(s), then regression coefficients [58] can be used. However, if the outcome is categorical a more complex multivariate model with predictive probabilities should be used [58].

### Difficulties and limitations in the study

As this systematic review was not a review of interventions but of observational techniques, it posed some difficulties and limitations. Every effort was made to conduct this review to the highest standards as recommended in the Cochrane Handbook [78] and to report it using the MOOSE guidelines for reporting of systematic reviews and meta-analyses of observational studies [79]. These guidelines were used as there were no reporting guidelines for systematic reviews of cross sectional studies or of methodologies. There were several grey areas or areas not covered in these guidelines that were particularly difficult to overcome in this type of review, including;

• What databases and grey literature sources should be used in a review? Grey literature refers to literature that is not published in peer reviewed journals or books [78]

• How many reviewers are required at each of the searching, screening and appraisal stages?

• What critical appraisal tools should be used?

• Should the initial screening processes be carried out blind?

• How should grey literature sources be searched?

To overcome some of these problems we extensively searched numerous databases, including CAB Abstracts, to ensure maximum coverage of the veterinary literature as outlined by Grindlay et al. [80]. The lead author was familiar with pet population research therefore it was not possible to blind the screening process.

There are numerous critical appraisal tools that have been created to examine observational studies but they are focused mainly on case–control and cohort studies. There is a lack of critical appraisal tools available specifically aimed at cross sectional studies [81,82]. The authors designed their own critical appraisal tool specifically to appraise cross sectional studies as it was deemed necessary to overcome the limitations of other appraisal tools.

Google was used for grey literature searches as it is the most common method for searching the internet [83]. It is important to note that although Google advised that it had 643,000,000 hits available at the time of the study, it only allowed a maximum of 2,000 hits to be viewed. As a comparison, at the time of the study Yahoo only allowed 1,000 hits to be viewed, hence Google was chosen. Google also tailors its results to the location and preferences of the searcher [84], which results in bias as the grey literature searches on one computer in the UK will not necessarily result in the same hits as on another computer, or in another country. The Google search did lead to one further study being included in the review; for other reviews the value of searching the grey literature in this way should be assessed. Ideally an all-inclusive grey literature resource for veterinary information should be created, allowing more targeted investigation of non-journal material.

## Conclusions

### Implications for practice

Pet population estimates are still helpful in focusing strategies for providing veterinary care; however it is important to take into account the biases of the study and to adjust decisions appropriately. The results from the studies found in this review are not directly comparable due to the differences in methods. Industry decision makers should examine the differences between studies before adjusting their marketing strategies.

### Implications for research

It can be taken from the results of the critical appraisal in this review that careful study design to minimise bias is a necessity before executing a study to estimate pet populations. There is also a need for researchers to become familiar with reporting guidelines such as STROBE.

## Methods

The Cochrane Handbook for Systematic Reviews of Interventions [78] and the Meta-analysis of Observational Studies in Epidemiology (MOOSE) reporting guidelines [79] were used in the development, execution and reporting of this review.

### Literature searching

MD and DG (information specialist) designed the methods for searching the literature. Relevant studies were identified using the following online bibliographic databases (date search completed in brackets): CAB Abstracts (9th May 2011), Web of Science (9th May 2011), MEDLINE (10th May 2011), Embase (11th May 2011), PubMed (11th May 2011), BIOSIS Previews (14th July 2011), and Zoological Record (14th July 2011).The Google search engine was used to identify studies outside of the peer - reviewed journal literature (13th May 2011).

#### Search strategy for identification of studies

Each bibliographic database and the Google search engine was systematically searched using the following search terms, or derivatives of these, depending on the subject heading terms used by the databases: (census OR censuses OR demography OR population OR “population density” OR “population dynamics”) AND (canidae OR canine OR canis OR dog OR dogs OR felidae OR feline OR felis OR cat OR cats OR pet OR pets). See Additional file 1 for full details of the searches used in each database.

#### Data collection

The searches were carried out by the first author (MD) and the results extracted and imported into the bibliographical software EndnoteX4 (Thomson Reuters) for the database searches and Microsoft Excel for the Google search. Endnote was used to automatically remove any duplicates from database searches by matching title and author. The dataset was then visually scanned by MD and any duplicates not found by Endnote were identified and removed. Articles that did not meet the inclusion criteria or met the exclusion criteria, assessed firstly by their title, and secondly by their abstract, were removed by MD, as shown in the flow chart in Figure 1. Translators were used to translate papers published in languages other than English. Initially just the materials and methods were translated to assess those papers that met the inclusion criteria.

The Google dataset was visually scanned and any duplicates identified and removed. Web page titles that did not meet the inclusion criteria were removed, and if their content clearly met the exclusion criteria they were removed by MD as shown in the flow chart in Figure 2.

The full text was then obtained, if possible, for the articles and web pages that remained from the online databases and Google searches. Articles were accessed through the internet if access was available from The University of Nottingham. If an article was unavailable online, an attempt was made to retrieve the article from The University of Nottingham Library or through the British Library Document Supply Centre. For the articles published in languages other than English, the remaining parts of the articles were then translated into English.

### Inclusion criteria/Exclusion criteria

The remaining articles were checked independently by MD and MB and included in the final analysis if they met all five of the following inclusion criteria:

• The studies concerned domestic dogs (*Canis lupus familiaris*) and/or cats (*Felis catus*);

• The studies examined owned or pet dogs or cats, or used the WHO/WSPA guidelines on dog/cat classifications [85];

• The studies provided an estimate of the size of the owned dog or cat population;

• The studies collected raw data on dog and cat ownership;

• The studies analysed primary data.

Articles that then remained were excluded from the final analysis if they met either of the following exclusion criteria:

• The studies used modelling to estimate the population without generating raw data;

• The studies were a review or summation of another study.

If two papers were published based on the same data, the earliest version of the paper was included and the later one excluded, unless extra information was available in the more recent publication. If a conference abstract and a peer reviewed publication were found pertaining to the same study, the peer reviewed publication was included and the conference abstract excluded.

An additional, final inclusion criterion required that the methods for calculating the population size estimate reported in the study were transparent and repeatable. If the methods for calculating the estimated population size were not deemed sufficient for readers to reproduce the calculation, the study was excluded (independently by MD and MB). If the study failed to describe the methods fully two attempts on separate occasions were made to contact the authors for further information. If contact details were not found, contact was unsuccessful or the required information was not obtained the study was excluded from the review at this stage.

In the event of disagreement regarding inclusion of a study, the study was read in full and resolved by consensus (MD and MB). If there was still uncertainty after this point, a third reviewer (RD) assessed the study independently and a decision was made by consensus or majority vote.

### Critical appraisal

The authors designed their own critical appraisal tool specifically to appraise cross sectional studies and piloted it within the School of Veterinary Medicine and Science at The University of Nottingham. The criteria examined by the critical appraisal tool included appropriateness of study design, representativeness of sample frame, selection process, analytical methods, completeness of description of methods, internal consistency of results, completeness of discussion and justification of study conclusions.

Critical appraisal of studies that fulfilled both the inclusion and exclusion criteria was carried out independently by MD and MB. Studies were excluded if they were deemed of insufficient quality based on the critical appraisal results by consensus between MD and MB. If there was disagreement, a third reviewer (RD) assessed the study independently and a decision was made by consensus. In the cases where additional information was required from the author and adequate information was obtained, it was the original paper that was critically appraised with the additional information taken into consideration. The critical appraisal process was used to identify areas of possible biases in the included studies.

### Data summary

After critical appraisal, a summary of findings for the included studies was produced by MD. The studies were grouped according to the data collection methods used and the analytical methods used to estimate the cat and dog population. The potential biases (Table 5) associated with these methods were examined.

**Table 5:** The definition of potential biases as they affect pet ownership studies

**Type of bias**	**Definition**
Selection bias	Selection bias is a systematic error that occurs when the distribution of factors associated with pet ownership in the target population differs from those in the study population [58].
Non-response bias	Non-response bias is when the characteristics that are associated with pet ownership of respondents differ from the characteristics of those that did not respond [66].
Measurement bias	Measurement bias is caused by inaccurate responses to survey questions which can result in misclassification of pet owners [66].
Length of sampling bias	Biases introduced by length of sampling time are introduced by estimating point prevalence (number of owned pets) over a relatively long sampling time, or by using period prevalence (number of owned pets in a given time period) to estimate point prevalence [86].

## Abbreviations

AVMA: The American Veterinary Medical Association; MOOSE: Meta-analysis of observational studies in epidemiology; PFMA: Pet Food Manufacturers Association; STROBE: The Strengthening the Reporting of Observational Studies in Epidemiology; UK: The United Kingdom; USA: The United States of America.

## Competing interests

The authors declare that they have no competing interests.

## Authors’ contributions

MD participated in the design of the study, designed and carried out the searching of the literature, collected and analysed the data, and drafted the manuscript. RD participated in the design of the study, analysed the data, and helped to draft the manuscript. JS participated in the design of the study and helped to draft the manuscript. VA participated in the design of the study and helped to draft the manuscript. DG participated in the design of the literature searches and helped to draft the manuscript. MB participated in the design of the study, designed and carried out the searching of the literature, collected and analysed the data and helped to draft the manuscript. All authors read and approved the final manuscript.

## Supplementary Material

Additional file 1 Search strategy for identification of studies in a systematic review examining methods to estimate owned dog and cat populations. Click here for file
