# Supplementary material for: Methods used to estimate the size of the owned cat and dog population: a systematic review
Source: BMC Vet Res. 2013 Jun 19;9:121. doi: 10.1186/1746-6148-9-121 (PMC3689088; doi:10.1186/1746-6148-9-121)
Supplement: Additional file 1 — Search strategy for identification of studies in a systematic review examining methods to estimate owned dog and cat populations. [file 1746-6148-9-121-S1.pdf]

Search strategy for identification of studies in a systematic review examining methods to estimate owned dog and cat populations

BIOSIS Previews® (1969-present),

Topic=(Cat OR Cats OR Felidae or Felis or Feline OR Dogs OR Dogs OR Canine OR Canidae OR Canis OR Pets OR Pet) AND (Population OR Census OR Demographics OR Population Dynamics OR population control OR population density OR population groups OR population growth OR population surveillance)

Web of ScienceSM (1899-present),

Topic=(Cat OR Cats OR Felidae or Felis or Feline OR Dogs OR Dogs OR Canine OR Canidae OR Canis OR Pets OR Pet) AND (Population OR Census OR Demographics OR Population Dynamics OR population control OR population density OR population groups OR population growth OR population surveillance)

Zoological Record® (1978-present):

Topic=(Cat OR Cats OR Felidae or Felis or Feline OR Dogs OR Dogs OR Canine OR Canidae OR Canis OR Pets OR Pet) AND (Population OR Census OR Demographics OR Population Dynamics OR population control OR population density OR population groups OR population growth OR population surveillance)

PubMed:

(census OR censuses OR demography OR population OR population density OR population dynamics) AND (canidae OR Canine OR canis OR dog OR dogs OR cat OR cats OR felidae OR feline OR felis OR pet OR pets)

Google:

(census OR censuses OR demography OR population OR "population density" OR "population dynamics") AND (canidae OR Canine OR canis OR dog OR dogs OR cat OR cats OR felidae OR feline OR felis OR pet OR pets)

MEDLINE (Ovid SP) (1948–present):

1. Population.mp.
2. exp Population/
3. exp Animal Population Groups/
4. exp Population Density/
5. exp Population Dynamics/
6. Population density.mp.
7. population dynamics.mp.
8. demography.mp.
9. exp Demography/
10. Census.mp.
11. exp Censuses/
12. 1 or 2 or 3 or 4 or 5 or 6 or 7 or 8 or 9 or 10 or 11
13. Cats.mp. 14. cat.mp. 15. exp Cats/
16. feline.mp. 17. exp Felis/
18. exp Felidae/
19. felis.mp. 20. felidae.mp.
21. 13 or 14 or 15 or 16 or 17 or 18 or 19 or 20
22. DOG/
23. dogs.mp.
24. dog.mp.
25. Canidae.mp.
26. Canis.mp.
27. Canine.mp.
28. exp CANIDAE/
29. 22 or 23 or 24 or 25 or 26 or 27 or 28
30. 21 or 29
31. pet.mp. or exp pets/
32. pets.mp. 33. 30 or 31 or 32

Embase (Ovid SP) (1980–present):

1. Cat.mp.
2. Cats.mp.
3. exp CAT/
4. felis.mp.
5. felidae.mp.
6. feline.mp.
7. exp FELIDAE/
8. 1 or 2 or 3 or 4 or 5 or 6 or 7
9. Population.mp.
10. exp POPULATION/
11. exp population density/
12. Population density.mp.
13. Population dynamics.mp.
14. exp population dynamics/
15. Demography.mp.
16. exp DEMOGRAPHY/
17. census.mp.
18. exp population research/
19. 9 or 10 or 11 or 12 or 13 or 14 or 15 or 16 or 17 or 18
20. DOG/
21. dogs.mp.
22. dog.mp.
23. Canidae.mp.
24. Canis.mp.
25. Canine.mp.
26. exp CANIDAE/
27. 20 or 21 or 22 or 23 or 24 or 25 or 26
28. 8 or 27
29. pets.mp. or exp pet animal/
30. pet.mp. 31. 28 or 29 or 30
32. 19 and 31

<u>CAB abstracts (Ovid SP) (1948–present):</u>

1. Cats.mp.
2. Cat.mp.
3. exp cats/
4. Felis.mp.
5. Felidae.mp.
6. feline.mp.
7. exp Felis/
8. exp Felidae/
9. 1 or 2 or 3 or 4 or 5 or 6 or 7 or 8
10. Dog.mp.
11. Dogs.mp.
12. exp dogs/
13. Canidae.mp.
14. canis.mp.
15. canine.mp.
16. exp Canidae/
17. exp Canis/
18. 10 or 11 or 12 or 13 or 14 or 15 or 16 or 17
19. exp Pets/
20. pets.mp.
21. pet.mp.
22. 19 or 20 or 21
23. exp populations/
24. Population.mp.
25. Population Density.mp.
26. exp population density/
27. exp population dynamics/
28. Population dynamics.mp.
29. Demography.mp.
30. exp demography/
31. exp censuses/
32. census.mp.
33. 23 or 24 or 25 or 26 or 27 or 28 or 29 or 30 or 31 or 32 or 33
34. 9 or 18 or 22 or 33
